# Supplementary material for: MD Simulation Reveals Regulation of Mechanical Force and Extracellular Domain 2 on Binding of DNAM-1 to CD155
Source: Molecules. 2023 Mar 21;28(6):2847. doi: 10.3390/molecules28062847 (PMC10053669; doi:10.3390/molecules28062847)
Supplement: Supplementary file 1 [file molecules-28-02847-s001.zip › molecules-2280004-supplementary.pdf]

molecules

Supplementary Materials

MD Simulation Reveals Regulation of Mechanical Forceand Extracellular Domain 2 on Binding of DNAM-1 to CD155

Liping Fang, Yang Zhao, Pei Guo, Ying Fang * and Jianhua Wu *

Institute of Biomechanics/School of Biology and Biological Engineering, South China University of Technology, Guangzhou 510006, China
* Correspondence: yfang@scut.edu.cn (Y.F.); wujianhua@scut.edu.cn (J.W.)

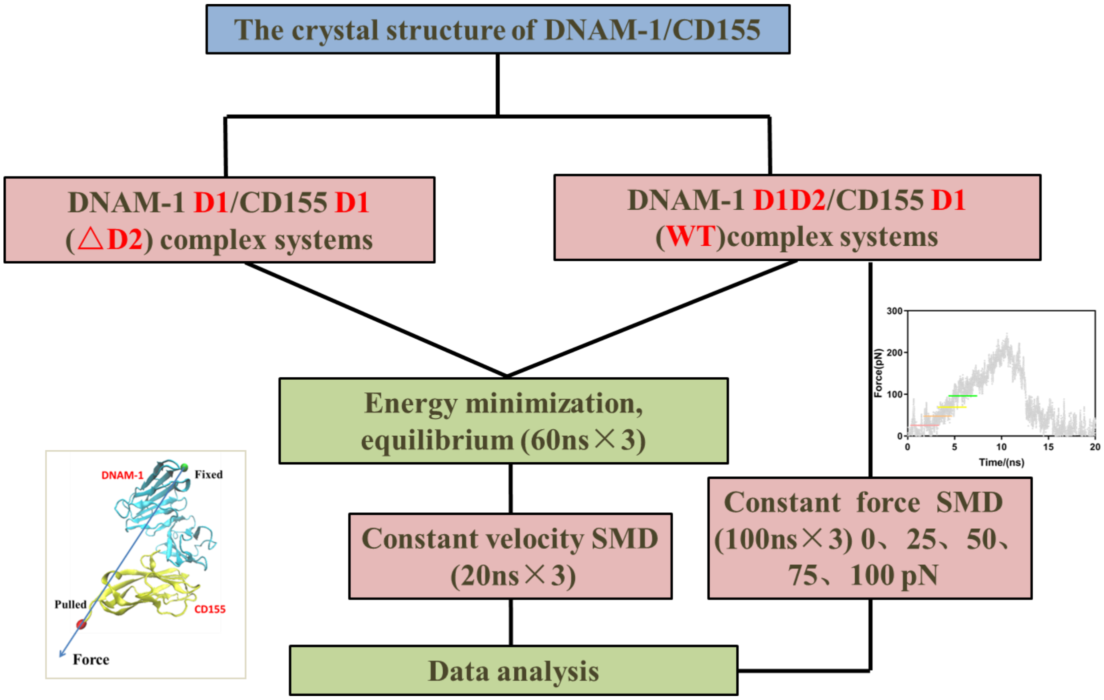

Figure S1. The ensemble workflow of computational procedure.

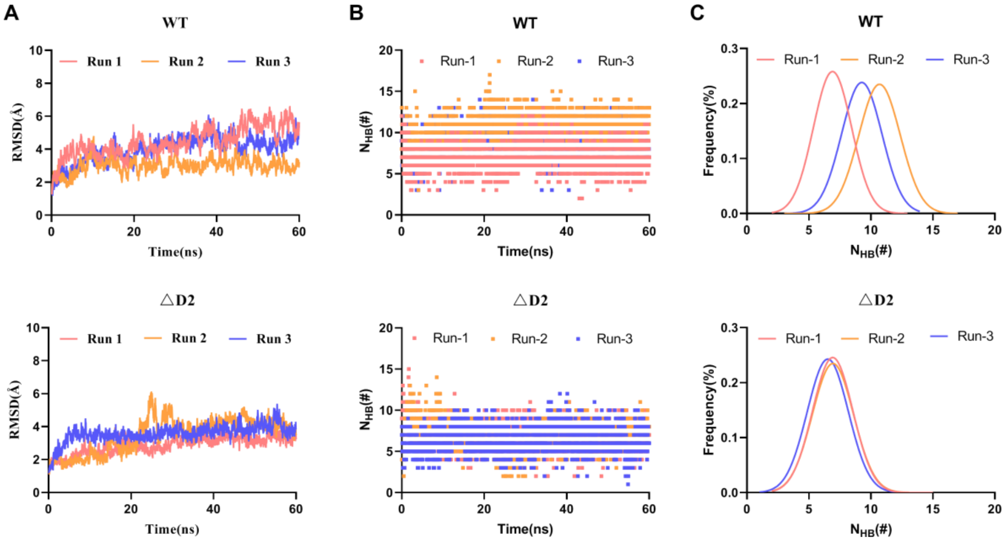

Figure S2. The time courses of root mean square deviation (RMSD) of heavy atoms and the number of H-bonds(NHB) be-tween the interfaces, and the distributions of H-bonding events within 60 ns at binding site of the two complexes (WT and △D2) for thrice 60 ns equilibriums. (A) The time courses of RMSD, (B) the time courses of NHB,and (C)the gauss frequen-cies of NHB of for three runs with the two complex.

Table S1. Residue interaction on binding site of DNAM-1/CD155 complex.

No	Residue name		Force(pN)				
	DNAM-1	CD155	0pN	25pN	50pN	75pN	100pN
1	T46	Q63	0.85±0.02	0.87±0.04	0.89±0.02	0.87±0.01	0.83±0.06
2	Q47	T127	0.80±0.01	0.77±0.01	0.74±0.04	0.76±0.05	0.76±0.05
3	E49	S132	0.86±0.04	0.78±0.09	0.80±0.09	0.80±0.05	0.77±0.09
4	Q115	S74	0.65±0.05	0.60±0.09	0.58±0.06	0.58±0.04	0.63±0.04
5	Q47	S62	0.80±0.05	0.73±0.03	0.69±0.2	0.78±0.04	0.72±0.15
6	R72	E57	0.54±0.03	0.68±0.17	0.68±0.22	0.54±0.23	0.20±0.24
7	S59	R133	0.22±0.09	0.17±0.02	0.18±0.02	0.25±0.11	0.06±0.06
8	Y113	G83	0.52±0.06	0.48±0.12	0.40±0.09	0.36±0.16	0.41±0.03
9	T112	Q63	0.44±0.07	0.51±0.08	0.53±0.12	0.49±0.04	0.49±0.07
10	K190	E71	0.39±0.06	0.38±0.15	0.29±0.28	0.38±0.13	0.34±0.18
11	Y111	S132	0.10±0.01	0.15±0.14	0.10±0.18	0.25±0.02	0.15±0.17
12	Q119	E71	0.33±0.10	0.40±0.20	0.33±0.28	0.40±0.10	0.39±0.18
13	R72	P129	0.29±0.09	0.19±0.16	0.13±0.22	0.23±0.13	0.32±0.20
14	R72	Q130	0.11±0.06	0.15±0.14	0.11±0.19	0.18±0.09	0.31±0.13

Table S2. Residue interaction on interface of DNAM-1 D1 and D2.

No	Residue name		Force(pN)				
	D1	D2	0pN	25pN	50pN	75pN	100pN
1	S26	I173	0.97±0.01	0.95±0.04	0.96±0.04	0.98±0.00	0.98±0.01
2	T25	D174	0.56±0.11	0.57±0.08	0.57±0.08	0.54±0.04	0.62±0.01
3	D128	R221	0.05±0.09	0.83±0.20	0.64±0.20	0.91±0.01	0.82±0.14
4	E36	R171	0.45±0.27	0.65±0.15	0.60±0.15	0.58±0.13	0.79±0.02
5	Q119	K190	0.17±0.15	0.25±0.26	0.25±0.26	0.19±0.13	0.31±0.07
6	H24	L175	0.45±0.39	0.54±0.23	0.51±0.23	0.67±0.01	0.50±0.15
7	H24	K190	0.15±0.13	0.07±0.17	0.18±0.17	0.17±0.09	0.15±0.12
8	E19	R185	0.28±0.24	0.12±0.07	0.20±0.07	0.33±0.06	0.18±0.17
9	K120	D174	0.21±0.20	0.22±0.24	0.25±0.24	0.04±0.04	0.25±0.07
10	E36	Q172	0.35±0.29	0.44±0.24	0.55±0.24	0.31±0.05	0.33±0.09
11	E20	R185	0.27±0.17	0.19±0.43	0.30±0.43	0.22±0.18	0.16±0.14
